# A cross sectional study on factors associated with harmful traditional practices among children less than 5 years in Axum town, north Ethiopia, 2013

**DOI:** 10.1186/1742-4755-11-46

**Published:** 2014-06-21

**Authors:** Kahsu Gebrekirstos, Mesfin Abebe, Atsede Fantahun

**Affiliations:** 1 Department of Nursing, College of Health Sciences, Mekelle University, Mek’ele, Ethiopia; 2 Department of nursing, College of health Sciences, Addis Ababa University, Addis Ababa, Ethiopia

**Keywords:** Harmful tradition practice, Uvula cutting, Milk teeth extraction, Eye brow incision

## Abstract

**Background:**

Every social grouping in the world has its own cultural practices and beliefs which guide its members on how they should live or behave. Harmful traditional practices that affect children are Female genital mutilation, Milk teeth extraction, Food taboo, Uvula cutting, keeping babies out of exposure to sun, and Feeding fresh butter to new born babies. The objective of this study was to assess factors associated with harmful traditional practices among children less than 5 years of age in Axum town, North Ethiopia.

**Methods:**

Community based cross sectional study was conducted in 752 participants who were selected using multi stage sampling; Simple random sampling method was used to select ketenas from all kebelles of Axum town. After proportional allocation of sample size, systematic random sampling method was used to get the study participants. Data was collected using interviewer administered Tigrigna version questionnaire, it was entered and analyzed using SPSS version 16. Descriptive statistics was calculated and logistic regressions were used to analyze the data.

**Results:**

Out of the total sample size 50.7% children were females, the mean age of children was 26.28 months and majority of mothers had no formal education. About 87.8% mothers had performed at least one traditional practice to their children; uvula cutting was practiced on 86.9% children followed by milk teeth extraction 12.5% and eye borrows incision 2.4% children. Fear of swelling, pus and rapture of the uvula was the main reason to perform uvula cutting.

**Conclusion:**

The factors associated with harmful traditional practices were educational status, occupation, religion of mothers and harmful traditional practices performed on the mothers.

## Background

According to the definition adopted by the World Health Organization (WHO) in 1978, Traditional Medicine is “The sum total of all the knowledge and practices, whether explicable or not, used in diagnosis, prevention and elimination of physical, mental or social imbalance and relying exclusively on practical experience and observation handed down from generation to generation whether verbally or in writing” [1,2]. Every social grouping in the world has its own cultural practices and beliefs which guide its members on how they should live or behave [3]. The list of harmful practices worldwide is long, ranging from lesser-known practices such as uvula cutting and milk teeth extraction, forced feeding and nutritional taboos, to the more commonly known practice female genital mutilation/cutting (FGM) [4]. The basic question whether a practice is harmful or necessary is often hotly debated that sometimes rely on simplistic divisions between “Western” and local medical values [5]. All social groups over the world have specific practices and beliefs which often have strong cultural underpinnings. These can be positive but they can also be negative [6].

Harmful practices based on tradition, culture, religion or superstition are often perpetrated against very young children or infants, who are clearly lacking the capacity to consent or to refuse consent themselves [7]. Apart from language, each ethnic group strives to maintain as an indicator of origin and identity, there are certain cultural practices that are unique to certain groups. Some of the practices impact negatively on the enjoyment of human rights in general and the rights of women and children in particular [8]. The Convention on the Rights of the Child prohibits traditional practices harmful to the health of children [9]. Traditional medical and behavioral practices in sub- Saharan Africa have been evaluated infrequently in relation to risk of infectious disease transmission [10].

Ethiopia has beneficial traditional practices such as breast feeding, settling quarrels, social gathering and others that can be examples to the external world. On the other hand, there are harmful traditional practices (HTPs) that affect the health and social well-being of women and children [11]. HTPs that affect children are FGM, Milk teeth extraction (MTE), Food taboo, Uvula cutting (UC), keeping babies out of exposure to sun, and Feeding fresh butter to new born babies [12]. The National constitution, Criminal and Family law’s, Education, Health, Population and Cultural polices of Ethiopia included articles that directly or indirectly combat HTPs. However, their existence alone does not provide a guarantee of protection to the affected communities [13]. The Ethiopian demographic health survey (EDHS) 2005 showed that the prevalence of uvulectomy or tonsillectomy increases with woman’s age rising from 37% (age 15–19) to 49% (age 45–49), indicating an increasing trend in the practice. The practice is most prevalent among rural women, women residing in Tigray, women with no education, and women in the lowest wealth [14]. Based on the baseline survey (BLS) on HTPs in Ethiopia in 1997, the main reasons listed for uvula cutting were: swelling, pus which finally erupts leading to death (53.1% on BLS and 21.7% follow up); no better cure in modern medicine (13.5 on BLS and 19.8% Follow up) and prevent repeated sore throat (15.6% on BLS and 14.2% in follow up) [15]. The reasons to perform MTE mentioned by mothers were: To prevent diarrhea and/or vomiting, fever, problems of growth and development, root of milk teeth can have things such as worms or hair. The only reason of eye borrow incision was believed to prevent eye diseases or infections [15,16].

In Ethiopia few studies have been conducted to assess the occurrence of different HTPs performed specifically on children. The significance of this study was to assess factors associated with HTPs among children in Axum town North Ethiopia. It was also aimed to address additional issues to fight the practices since they are still ongoing.

## Methods

Community based cross sectional study was conducted was conducted from June 5 - 30/2013 in Axum town, north Ethiopia in 752 mothers who have children less than 5 years. The sample size was calculated using single proportion formula with the prevalence of uvula cutting in Tigray region (P = 66.4%) which give maximum number, confidence level of 95% and 5% significance level. The study participants were mothers who have children less than 5 years because the literatures showed that they are at frontline for child care. Multi stage sampling was used to reach to the study participants; lottery method was employed to select the ketenes (districts) and after proportional allocation to each ketena systematic sampling method was used to select the study participants. Structured questionnaire adapted from the Follow up survey of HTPs in Ethiopia by NCTPE, 2008 was used to collect data. It was first prepared in English and translated to local language (Tigrigna). Data was collected using interview for 1 week. To ensure quality of data pre test was conducted in 10% of sample size at similar population which were not included in the study. Data was checked and cleaned also daily for completeness and consistency during data collection. Data was entered and analyzed using SPSS software (version 16.0). Multiple logistic regressions were used to analyze the relationship between the dependent and independent variables as well as associations was done using odds ratio. Tables and graphs were used to present the results. The proposal was submitted to Addis Ababa University, Department of Nursing and Midwifery Institutional Review Board, for approval. Following approval, official letter of cooperation was written to concerned bodies. Study participants were informed about the purpose, advantage and disadvantage of the study. No personal identifiers were used and participants had the right to refuse at any stage of interview. Informed verbal consent was obtained from each mother prior to interview. Confidentiality was assured for all the information provided.

## Results

### Socio demographic characteristics

In this study a total of 752 mothers who had children less than five years were interviewed with response rate of 100%. Number of female children was 381(50.7%) and the mean age of children was 26.28 months (SD = + 15.98, Range 1–59 months) while mean age of mothers was 30.55 years (SD = + 6.22, range 19–51 years). Majority of the respondents, 611(81.2%) were Orthodox Christian and the rest were Muslims in religion. Regarding occupational status about 588 (78.2%) of respondents had no work and 6 (0.8%) had other works like local cloths makers, local drink (Tella) makers and beauty salon. 379 (50.4%) attended primary school, 196(26.1%) in secondary school, 100(13.3%) illiterate. Majority of the respondents had less than 500 birr monthly income as well as the rest had more than 500 birr (Table 1).

**Table 1:** Socio demographic characteristics of children less than five years in Axum town, north Ethiopia, 2013

**Variable**		**Frequency (n = 742)**	**Percent**
Sex	Male	371	49.3
	Female	381	50.7
Age of child in months	0-4	47	6.2
	5-9	92	12.2
	10-14	107	14.2
	15-19	51	6.8
	20-24	87	11.6
	25-29	40	5.3
	30-34	62	8.2
	35+	266	35.4
Age of mothers	15-19	6	0.8
	20-24	115	15.3
	25-29	222	29.5
	30-34	168	22.3
	35-39	159	21.1
	40-44	71	9.4
	45+	11	1.5
Religion	Orthodox	611	81.2
	Muslim	141	18.8
Occupation	Jobless	588	78.2%
	Civil servant	56	7.4
	Merchant	98	13.0
	Farmer	4	0.5
	Others*	6	0.8
Ethnic group	Tigraway	748	99.5
	Amhara	4	0.5
Educational Status	Illiterate	100	13.3
	Religious	17	2.3
	Primary school	379	50.4
	Secondary school	196	26.1
	Higher education	60	8.0
Monthly income	lessthan500	492	65.4
	501-1000	130	17.3
	greaterthan1000	130	17.3

*Others = local cloths makers, beauty salon, local drinks makers.

### Prevalence of harmful traditional practices

Out of the 752 respondents 746(99.2%) had information on at least one harmful traditional practices in which 301 (40%) of them had information on all of the mainly recognized HTPs (uvula cutting, milk teeth extraction, FGM and eye borrow incision), 108(14.4%) about uvula cutting and 69 (9.2%) about female genital mutilation. From the total number of participants 660(87.8%) had performed at least one HTPs on their children. Among the HTPs performed on children, uvula cutting was practiced on 654 (86.9%) children followed by milk teeth extraction 95(12.5%) children and eye borrows incision 18(2.4%) children (Table 2).

**Table 2:** Harmful traditional practices among children less than five years in Axum town, North Ethiopia, 2013

**Variable**		**Frequency (n)**	**Percent (%)**
**Information about HTPs**	Yes	746	99.2
	No	6	0.8
Information by type of HTPs*	Uvula cutting	588	78.2
	Female genital mutilation (FGM)	493	65.5
	Milk teeth extraction (MTE)	380	50.5
	Eye borrow incision	362	48.1
	Bloodletting	308	40.9
Source of information*	Mass media	314	41.7
	Health personnel	410	54.5
	Family members	372	49.5
	Meeting	127	16.9
	Others**	4	0.5
**Any HTPs performed on mother**	Yes	618	82.2
	No	134	17.8
Type of HTPs performed on mother*	Uvula cutting	599	79.6
	Female genital mutilation	5	0.7
	Milk teeth extraction	43	5.7
	Eye borrow incision	116	15.4
	Bloodletting	16	2.13
**HTPs performed on children**	Yes	660	87.8
	No	92	12.2
Types of HTPs performed on children*	Uvula cutting	654	86.9
	Milk teeth extraction	94	12.5
	Eye borrow incision	18	2.4

*more than one answer was given.

**others = school.

### Reasons and factors associated with harmful traditional practices

**Figure 1:**
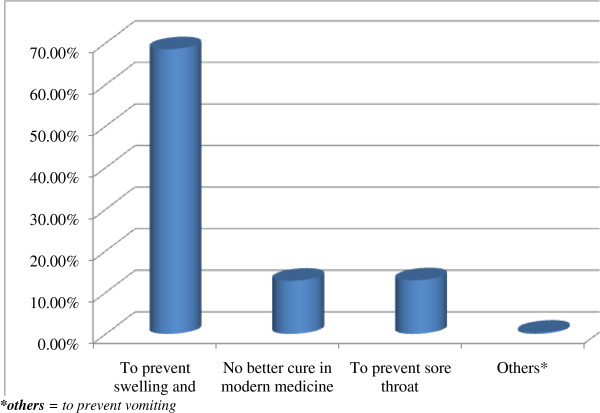
Distribution of reasons to perform uvula cutting among children less than 5 years in Axum town, North Ethiopia, 2013.

The main reason to perform uvula cutting mentioned by mothers was to prevent swelling, pus and rapture of the uvula which can lead the child to death mentioned by 515(68.5%) mothers. Other reasons were no better medical cure and to prevent sore throat mentioned by 96(12.8%) and 97(12.9%) respondents respectively (Figure 1).

Out of the respondents who practice milk teeth extraction, 62(8.2%) reason out to prevent diarrhea and vomiting; prevention of teething problem and cure or prevention diseases were other reasons. All mothers who practice eye borrow incision 18 (2.4%) explain only one reason; to treat eye diseases. Majority of the mothers 484(64.4%) think traditional practices as harmful; of which 168(22.3%) respond to all HTPs. More than half of the mothers 403(53.6%) explained that they will perform the traditional practices especially uvula cutting in the future. About 403(53.6%) mothers didn’t support traditional practices especially uvula cutting to be eradicated; their main reasons were that “HTPs mainly uvula cutting is beneficial” and “eradication is against culture” (Table 3).

**Table 3:** Reasons associated with harmful traditional practices among children less the 5 years in Axum town north Ethiopia, 2013

**Variable**		**Frequency (n = 742)**	**Percent (%)**
Reasons to perform MTE*	Prevent diarrhea and vomiting	62	8.2
	Prevent problems of growth	5	0.7
	development		
	Root of teeth grows worms	21	2.8
	MTE prevents or cures disease	9	1.2
	Prevents teething problems	8	1.1
**Reason to perform Eye borrow incision**	Treatment of eye disease	18	2.4
**Thinking if HTPs are harmful**	Yes	484	64.4
	No	268	35.6
Which HTPs are harmful*	FGM	472	62.7
	Eye borrow incision	338	44.9
	bloodletting	336	44.7
	Milk teeth extraction	315	41.9
	Uvula cutting	168	22.3
**Why HTPs are not harmful**	The child will be harmed	213	28.3
	Previous experience of child death because of HTPs	25	3.3
	It is our culture	30	4.0
**Will you perform in the future**	Yes	403	53.6
	No	349	46.4
**Should HTPs eradicated**	Yes	349	46.4
	No	403	53.6
**Why HTPs shouldn’t eradicated**	Beneficial Against culture	367	48.8
	Against culture	36	4.8

* = *More than one response was given.*

Assessment of association of variables was calculated using with multivariate logistic regression, Orthodox christian mothers practiced HTPs 2 times as compared with Muslim mothers (AOR = 2.113(1.079-4.140), CI = 95%). There was strong association also between occupation and practice of HTPs on children. As a result jobless or house wife mothers practiced harmful traditional practices 5 times as compared with those who had occupation(AOR = 5.319(1.517-18.657), CI = 95%). Regarding educational status, Mothers who had no formal education practices HTPs 4 times than educated mothers (AOR = 3.913(1.262-12.130), CI = 95%).

Multiple logistic regressions showed very strong association between HTPs performed on the mothers themselves and practice of HTPs to their children. Those mothers who had traditional practices performed on themselves practiced HTPs on their children 24 times than those who didn’t have any HTPs performed on them (AOR = 24.890(13.853-44.719), CI = 95%) (Table 4).

**Table 4:** Socio demographic and maternal practice versus practice of HTPs on children among children less than 5 years in Axum town, north Ethiopia, 2013

**Variable**	**Practice of HTPs on children**		**COR (95% C.I)**	**AOR (95% C.I)**
	**Yes**	**No**		
**Sex**				
**Male**	329(49.8%)	42(45.7%)	0.845(0.546-1.309)	1.314(0.757-2.281)
**Female**	331(50.25)	50(54.3%)	1.0	1.0
**Age of mother**				
**Less than 25**	99(15.0%)	22(23.9%)	0.842(0.469-1.514)	1.180(0.554-2.512)
**25-29**	187(28.3%)	35(38.0%)	0.474(0.237-0.946)*	0.576(0.216-1.541)
**30-34**	152(23.0%)	16(14.4%)	0.385(0.199-0.744)**	0.668(0.244-1.829)
**35+**	222(33.6%)	19(20.7%)	1.0	1.0
**Marital Status**				
**Married**	555(84.1%)	105(15.9%)	1.0	1.0
**Not married**	83(90.2%)	9(9.8%)	1.745(0.50-3.580)	1.044(0.437-2.498)
**Religion**				
**Orthodox**	547(82.9%)	64(69.6%)	2.118(1.300-3.450)**	2.113(1.079-4.140)*
**Muslim**	113(17.15)	28(30.4%)	1.0	1.0
**Occupation**				
**Jobless/housewife**	531(80.5%)	57(62%)	3.105(1.599-6.030)**	5.319(1.517-18.657)**
**Civil servant**	42(6.4%)	14(15.2%)	2.249(1.298-3.895)**	2.473(0.997-6.137)
**Merchant & others**	87(13.2%)	21(22.8%)	1.0	1.0
**Ethnicity**				
**Tigrian**	657(99.5%)	91(98.9%)	2.407(0.248-23.382)	2.185(0.074-64.748)
**Amara**	3(0.5%)	1(1.1%)	1.0	1.0
**Educational status**				
**No formal education**	113(17.1%)	4(4.3%)	4.545(1.635-12.632)**	3.913(1.262-12.130)*
**Educated**	547(82.9%)	88(95.7%)	1.0	1.0
**In come**				
**Less than 500**	443(67.1%)	49(53.3%)	1.0	1.0
**500-1000**	114(17.3%)	16(17.4%)	0.422(0.252-.707)**	1.162(0.396-3.409)
**Greater than 1000**	103(15.6%)	27(29.3%)	0.535(0.273-1.050)	1.854(0.636-5.409)
**Number of children**				
**One to two**	305(42.6%)	58(63.0%)	1.0	1.0
**Three and above**	355(53.8%)	34(37.0%)	1.986(1.266-3.114)**	1.270(0.616-2.620)
**HTPs performed on mother**				
**Yes**	590(89.4%)	28(30.4%)	19.265(11.586 -32.035)***	24.890(13.853-44.719)***
**No**	70(10.6%)	64(69.6%)	1.0	1.0

*P < 0.05.

**p < 0.01.

***p < 0.001.

**COR** = Crude Odds Ratio.

**AOR** = Adjusted Odds Ratio.

**CI** = Confidence Interval.

## Discussion

The purpose of this study was to assess the factors associated with HTPs among children less than 5 years in Axum town, north Ethiopia.

All most all (99.2%) study participants had information about HTPs. This was higher than the Study conducted in SNNPR which showed that 65.3% the respondents had information about HTPs. Out of the mothers who had information , 86% of had information about Uvula cutting; this was higher as compared to follow up survey of NCTPE conducted in Ethiopia in 2008 which was 73.5% nationally and 79.8% in Tigray region. Information dissemination about FGM was 65.5% and about MTE 58.5% in this study. This was lower than the information dissemination reported by NCTPE in 2008 which was 73.8% and 69%nationally and 76.9% and 61.6% in Tigray region respectively. This difference might be because of time gap, the study area as well as sample size was smaller than the survey.

This study revealed that the leading HTP performed on children was uvula cutting (87%) this was lower than a study conducted in Dembia district, northwest Ethiopia which was practiced by 99.5% respondents. The variation might be because of time gap. But this was higher than the follow up survey of NCTPE in 2008 in which the prevalence of Uvula cutting was 66.4%. The second common HTP in this study was MTE which performed on 12.5% children but it was much lower than the follow up survey of NCTPE in 2008 that reported prevalence of milk teeth extraction 26.6%. This might be because of easily accessible medical service and awareness of families towards milk teeth extraction has been improved but not for uvula cutting. The third common HTP performed on children in this study was eyes borrow incision which was performed on 2.4% children, but it was all most null as compared to a study conducted in Dembia district, northwest Ethiopia [82%]. It might be due to fear of HIV/AIDS transmission as well as awareness of mothers towards modern medicine to treat eye infections or diseases have been improved. In this study there was no FGM practiced on children, it was in line with the study conducted in Dembia district, in which the practice of female circumcision was limited to only some areas and was supported by a small number of people. On the other hand the prevalence of FGM in Tigray region as was 21.2% in the follow up survey of NCTPE in 2008. The difference might be because awareness of families towards complications of FGM has been improved mainly with health education by extension workers, health facilities and mass media.

The main reason to perform Uvula cutting mentioned by 68.5% mothers was to prevent swelling, pus and rapture of the uvula which can lead the child to death. This was much higher than the same reason (21.7%) reported on the follow up survey of NCTPE in 2008. But in contrary to this study, a study conducted in Nigeria, 2011 suggested that a large majority of patients (65.5%) did not know the indication for uvula cutting being performed on them. This variation might be due to difference of cultural diversity of respondents. Other reasons to perform uvula cutting described by mothers were to prevent sore throat (12.9%) and no better cure in modern medicine (12.8%). This was in line and slightly higher than similar reasons mentioned on the follow up survey of NCTPE in 2008. But a study conducted in Nigeria, 2011 suggested throat pain as second common reason (21.8%) which was higher than this study. This difference might be similar to the above reason. Out of the respondents who practice MTE (12.5%) the reasons mentioned was to prevent diarrhea and vomiting (6.8%), this was in line with the reasons mentioned in the follow up survey in Ethiopia by NCTPE, 2008. This study was also in line with Study of HTPs in SNNPR, in 2005. This study showed eye borrow incision was performed to treat eye disease as mentioned by all mothers who perform it. This was also similar to the reasons listed in the follow up survey in Ethiopia by NCTPE, 2008.

In this study mothers who had no formal education practiced HTPs 4 times than educated mothers. This was similar with the study conducted in Dembia district, northwest Ethiopia which showed that as the level of education of respondents increased, the tendency towards not using the indicated traditional harmful health practices increased. It was also in line with the report from Ethiopian demographic health survey, 2005; the practice was most prevalent among women residing in Tigray, women with no education.

## Strength of the study

✓ The study tried to assess HTPs performed on children only and it can be used as a base line for further studies.

### Limitations of the study

✓ The study didn’t include qualitative method as a result factors associated with HTPs others than those factors described in the literatures may not be addressed

✓ The data collectors were urban health Extension workers (nurses) so mothers may be embarrassed to describe traditional practices like female genital mutilation.

### Implication for research and practice

As there are little studies conducted on HTPS, this study might have an implication for further research on HTPs and related topics because it can be used as a baseline data. It might also have an implication child health care; by identifying the gaps it help to reduce child morbidity and mortality and to improve quality of care.

## Conclusion

The study tried to assess the factors associated with harmful traditional practices among children less than five years in Axum town. Based on this study the following were concluded.

✓ The common HTPs performed on children in this study were uvula cutting, MTE and eye borrows incision. Uvula cutting was the leading HTP.

✓ As mentioned by mothers the main reason of uvula cutting was to prevent swelling, pus and rupture of the uvula which can lead the child to death.

✓ Prevention of diarrhea, vomiting and teething problem were main reasons described by mothers to perform milk teeth extraction.

✓ The only reason to practice eye borrow incision was to treat eye diseases or infections.

✓ The factors associated with HTPs were Educational status, occupation and religion of mothers as well as harmful traditional practices performed on the mothers.

## Abbreviations

BLS: Base line survey; EDHS: Ethiopian demographic health survey; FGM: Female genital mutilation; HTPs: Harmful traditional practices; MTE: Milk teeth extraction; NCTPE: National Committee for Traditional Practices of Ethiopia; SNNPR: Southern Nation Nationalities and Peoples Region; UC: Uvula cutting.

## Competing interest

The authors declare that they have no competing interests.

## Authors’ contribution

KG has made substantial contributions to conception and design, or acquisition of data, analysis and interpretation of data. AF has been involved in drafting the manuscript and revising it critically for important intellectual content. MA was the advisor of this research paper; He participated in revising and commenting the paper. All authors prepare and approved the final draft of the manuscript.

## Authors’ information

1. KAHSU GEBREKIRSTOS(BSc, MSc): Lecturer at Mekelle University, College of Health Sciences, department of Nursing, Mekelle, Tigray, Ethiopia.

2. MESFIN ABEBE(BSc, MSc): Lecturer at Addis Ababa University, College of health Sciences, department of nursing, Addis Ababa, Ethiopia.

3. ATSEDE FANTAHUN(BSc, MSc): Lecturer at Mekelle University, College of Health Sciences, department of Nursing, Mekelle Tigray, Ethiopia.
